# Electrochemical Sensor Based on Multi-Walled Carbon Nanotubes and N-Doped TiO_2_ Nanoparticles for Voltametric Simultaneous Determination of Benserazide and Levodopa

**DOI:** 10.3390/molecules27238614

**Published:** 2022-12-06

**Authors:** Elisangela Pacheco da Silva, Mayara da Silva Araujo, Marcos H. Kunita, Roberto Matos, Roberta Antigo Medeiros

**Affiliations:** 1 Department of Chemistry, State University of Maringá, Maringá 87020-900, PR, Brazil; 2 Department of Chemistry, State University of Londrina, Londrina 86057-970, PR, Brazil

**Keywords:** MWCNT, N-doped TiO_2_, glassy carbon modified electrode, Benserazide, L-dopa, square-wave voltammetry, electroanalytical method

## Abstract

An electrochemical sensor for simultaneous determination of Benserazide (BEZ) and levodopa (L-dopa) was successfully developed using a glassy carbon electrode (GCE) modified with multi-walled carbon nanotube and nitrogen-doped titanium dioxide nanoparticles (GCE/MWCNT/N-TiO_2_). Cyclic voltammetry and square wave voltammetry were employed to investigate the electrochemical behavior of different working electrodes and analytes. In comparison with unmodified GCE, the modified electrode exhibited better electrocatalytic activity towards BEZ and L-dopa and was efficient in providing a satisfactory separation for oxidation peaks, with a potential difference of 140 mV clearly allows the simultaneous determination of these compounds. Under the optimized conditions, linear ranges of 2.0–20.0 and 2.0–70.0 μmol L^−1^ were obtained for BEZ and L-dopa, respectively, with a limit of detection of 1.6 µmol L^−1^ for BEZ and 2.0 µmol L^−1^ for L-dopa. The method was applied in simultaneous determination of the analytes in pharmaceutical samples, and the accuracy was attested by comparison with HPLC-DAD as the reference method, with a relative error lower than 4.0%.

## 1. Introduction

**Figure 1:**
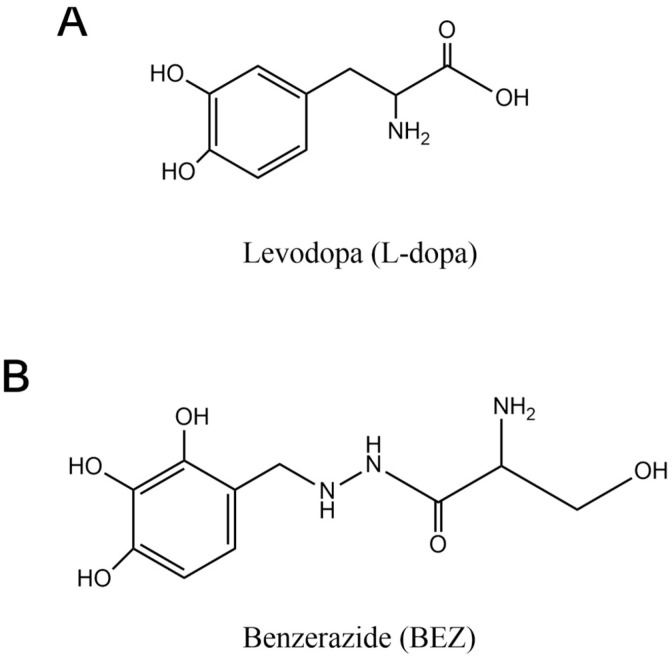
(**A**) L-dopa and (**B**) BEZ chemical structures.

Levodopa (L-dopa) ((2S)-2-amino-3-(3,4-dihydroxyphenyl) propanoic acid) and Benserazide (BEZ) (2-amino-3-hydroxy-N′-[(2,3,4-trihydroxyphenyl) methyl] propanehydrazide; hydrochloride) (Figure 1A,B) are drugs used together for the Parkinson’s disease treatment. L-dopa easily enters the central nervous system and is the immediate precursor of the neurotransmitter dopamine. However, for the treatment of diseases that are associated with depletion of dopamine in the brain, such as Parkinson’s disease, high doses of L-dopa are necessary because it is rapidly decarboxylated and very little unchanged drug is available to cross the blood–brain barrier for central conversion into dopamine. To increase the amount of L-dopa that enters the brain it is usually given with a peripheral decarboxylase inhibitor such as BEZ, thus permitting a considerably higher proportion of L-dopa to enter the brain [1,2]. Thus, the combination of L-dopa and BEZ is effective in the treatment of Parkinson’s, and its synergistic effect reduces the required dose of L-dopa for the optimal and earlier therapeutic response [3].

Some analytical methods have been developed for individual and simultaneous determination of L-dopa and BEZ in pharmaceutical and biological samples using mainly spectrophotometric [4,5,6] and chromatographic [7,8,9] techniques. However, in general, these procedures involve some extraction, the use of toxic solvents, and are highly time-consuming. Furthermore, for the spectrophotometric methods the use of chemometric tools was necessary for the simultaneous quantification of both analytes.

On the other hand, electrochemical techniques offer analytical options that are promising alternatives to spectrophotometric our chromatographic methods due to characteristics such as their relatively low operational cost, no organic solvents used, rapid and sensitive detection procedures that are suitable for faster analyses. Thus, some voltammetric methods have already been reported in the literature for the simultaneous determination of L-dopa and BEZ [10,11,12,13].

Zapata-Urzúa et al. [10] used a differential pulse voltammetry, a glassy carbon electrode (GCE), and a partial least-squares algorithm for the simultaneous determination of L-dopa, carbidopa, and BEZ in pharmaceutical formulations. Naushad et al. [11] reported on the simultaneous determination of BEZ and L-dopa in pharmaceutical tablets, human serum, and urine samples by differential pulse voltammetry using a poly(4-(2-pyridylazo)-resorcinol) modified GCE. Savan et al. [12] reported on the use of a GCE modified with multi-walled carbon nanotube (MWCNT) and poly(3-methylthiophene) films for simultaneous determination of the binary mixture of L-dopa and BEZ in the presence of ascorbic acid using voltammetric techniques. More recently, Miraki et al. [13] reported on the use of square wave analysis for the simultaneous determination of L-dopa and BEZ using as a working electrode a new modified carbon paste electrode amplified with NiO nanoparticle and n-methyl-3-butylimidazolium bromide.

MWCNTs are made of atomic layers of sp^2^-bonded carbon atoms and are a class of nanomaterials whose applicability in electrochemistry, especially in electroanalysis, is very well established [14,15,16,17]. There are two main effects generally observed when the MWCNTs are used as an electrochemical sensor, the reduction of the work potential due to the increase in electron transfer rate and the increase of the peak current signal due to the high electroactive surface area provided by the nanomaterial. These effects make it possible to detect analytes at a lower potential where interferences from concomitant compounds decrease and can improve the analytical parameters, such as sensitivity and detection limit [18,19].

Metallic nanoparticles, such as titanium dioxide (TiO_2_), also present attractive properties for electrochemical sensors as compared with other nanoparticles, such as fast electron transfer capability, large surface area, good biocompatibility and the synthesis is simple and low-cost. Furthermore, the TiO_2_ nanoparticles can increase the linear concentration ranges since the electrochemical response time is reduced because of the large adsorption capacity of target molecules [20,21,22,23].

The introduction of a non-metal element, such as a dopant, into TiO_2_ nanoparticle structure is common, they can improve the visible light photocatalytic and electrocatalytic activity of TiO_2_ since the charge transfer is induced by oxygen vacancy [24].

The nanoparticles of TiO_2_, doped or not, can be synthesized using different techniques, such as hydrothermal process and sol-gel [25,26,27]. However, both techniques can produce a large amount of residues, mainly solvents, which could have an environmental impact, polluting air and water. Supercritical carbon dioxide (scCO_2_) technology may be an alternative technique for the production of TiO_2_ nanoparticles and could considerably minimize environmental risks [28,29].

Additionally, the combination of CNTs with nitrogen-doped titanium dioxide (N-doped TiO_2_) can stabilize the response and improves the sensitivity and electrocatalytic properties [30].

Herein, the nitrogen-doped titanium dioxide (N-TiO_2_) synthesized by scCO_2_ and the MWCNTs functionalized were used as a nanocomposite (MWCNT/N-TiO_2_) to modify a GCE, which was used to develop an electroanalytical method for simultaneous determination of BEZ and L-dopa in pharmaceutical formulations.

## 2. Results and Discussion

### 2.1. Characterization of MWCNTs and MWCNTs/N-TiO_2_ Composite

**Figure 2:**
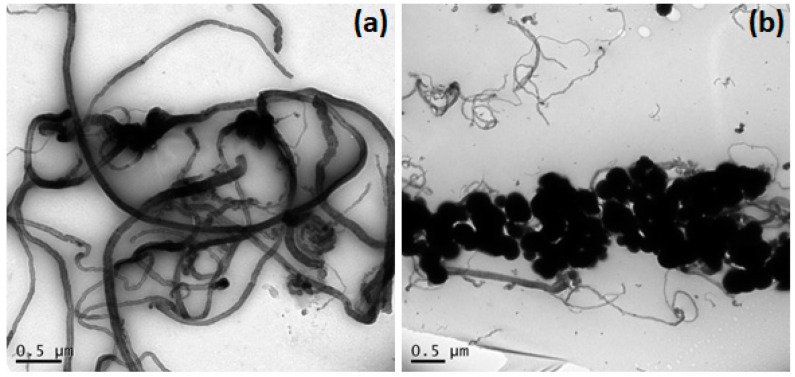
TEM images of MWCNT (**a**)and MWCNT/N-TiO_2_ (**b**).

Initially, the surface morphology of MWCNTs functionalized and MWCNTs/N-TiO_2_ were characterized by TEM. For analysis, MWCNTs and/or MWCNTs/N-TiO_2_ were dispersed in a chitosan solution (1% *m*/*v*) in acetic acid. As can be seen in Figure 2a, the MWCNTs are uniformly dispersed in the film of chitosan film. Furthermore, Figure 2b shows N-TiO_2_ interconnected nanoparticles, with an average size of around 200 nm, which adheres to the MWCNTs surface. The observed morphology is in agreement with those in MWCNTs/N-TiO_2_ previously reported by Huang et al. 2010 and Dehnav and Soleymanpour, 2021 [30,31].

### 2.2. Electrochemical Properties of Modified Electrode

**Figure 3:**
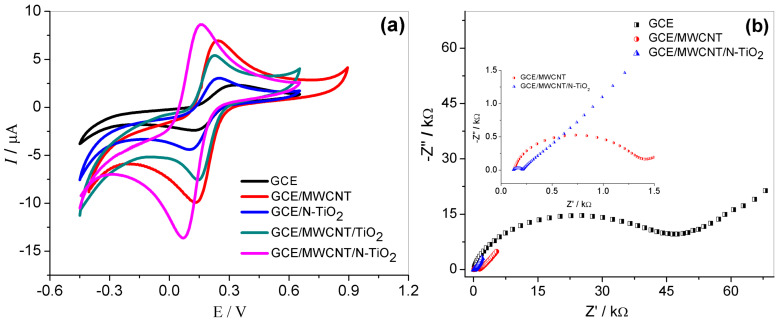
(**a**) Cyclic voltammograms obtained for 1.0 mmol L^−1^ Fe(CN)_6_^3−/4−^ solution in 0.1 mol L^−1^ KCl using different working electrodes: GCE, GCE /MWCNT, GCE/N-TiO_2_, GCE/WCNT/TiO_2_, GCE/MWCNT/N-TiO_2_, *v* = 50 mV s^−1^; (**b**) Nyquist plots obtained in the presence of 1.0 mmol L^−1^ Fe(CN)_6_^3−/4−^ solution in 0.1 mol L^−1^ KCl for GCE, GCE/MWCNT, and GCE/MWCNT/N TiO_2_.

Some experiments were carried out by CV and EIS in a solution containing 1.0 mmol L^−1^ of Fe (CN)_6_^3−/4−^. Cyclic voltammograms obtained from −0.6 to 1.2 V are displayed in Figure 3a. Well-defined reversible peaks are observed for all samples, which are directly related to the redox reaction of Fe (CN)_6_^3−/4−^. However, the highest redox peak current is noticed by GCE/MWCNT/N-doped TiO_2_, followed by GCE/MWCNT, GCE/MWCNT/TiO_2_, GCE/N-TiO_2_, and GCE, respectively. In addition, the ∆Ep value for GCE/MWCNT/N-TiO_2_ (∆Ep = 90 mV) is lower than GCE/MWCNT (∆Ep = 110 mV) and GCE (∆Ep = 209 mV), indicating that this electrode presents a high electron transfer rate when compared to others, the ΔE_p_ value is the closest to 60 mV, which is characteristic of a reversible redox system. Thus, the modification with MWCNT/N- TiO_2_ increased the electrochemical activity of the GCE.

The impedance spectra obtained at the formal potential of the [Fe(CN)_6_]^4−/3−^ redox couple using the GCE, GCE/MWCNT, and GCE/MWCNT/N-TiO_2_ show an excellent agreement. Figure 3b shows the Nyquist plots obtained by EIS. The technique is fast and precise to evaluate the surface resistance during the charge transfer process. For all electrodes studied, a semicircle in the high frequency was observed, which represents the electron transfer resistance (Ret), and a linear range in the low frequency related to the diffusion-controlled process [32]. The electron transfer resistances obtained were 49.0, 1.4, and 0.2 Ω for GCE, GCE/MWCNT, and GCE/MWCNT/N-TiO_2_, respectively. These results are in accordance with CV analysis and demonstrate the ability of electron transfer of Fe(CN)_6_^3−/4−^ on the MWCNT/N-TiO_2_ composite electrode. The better electrochemical performance achieved by GCE/MWCNT/N-TiO_2_ electrode compared to the others demonstrates that nitrogen doping to TiO_2_ nanoparticles enhances the intrinsic conductivity of the semiconductor and consequently improves the kinetics of the electrode. In addition, the high electroactive surface area of MWCNTs facilitates the diffusion electron in the electrolyte-electrode interface. Therefore, the synergic effect obtained with the nanocomposite MWCNT/N-TiO_2_ can accelerate the electron transfer rate, improving more sensibility of the system.

### 2.3. Electrochemical Behavior of BEZ and L-Dopa at GCE/MWCNT/N-TiO_2_

**Figure 4:**
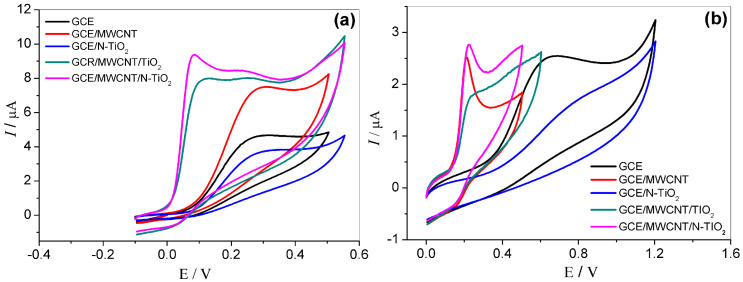
(**a**) Cyclic voltammograms obtained for 0.5 mmol L^−1^ of BEZ; (**b**) and 0.1 mmol L^−1^ of L-dopa with different working electrodes in 0.1 mol L^−1^ phosphate buffer solution (pH 7.0), *v* = 50 mV s^−1^.

CV was used to investigate the electrochemical response and the oxidation peak potential of BEZ and L-dopa. Figure 4 shows the cyclic voltammograms obtained from BEZ and L-dopa with the different working electrodes in 0.1 mol L^−1^ phosphate buffer solution (pH 7.0). As can be seen, only an oxidation peak for BEZ and L-dopa was observed, indicating an irreversible process for both. The voltammograms obtained with the bare GCE show peaks with good peak current intensity for both analytes. However, after the modification with MWCNT, the peak current increased for both, on the other hand, the oxidation potential declined only for BEZ, indicating an improvement in the interaction with the electrode surface, most likely caused by the interaction between the MWCNT negatively charged (zero charge potential ≈ 4.0) and the BEZ (pKa ≈ 8.0), whereas the pKa of L-dopa is ≈2.3 [33]. When the composites of MWCNT and TiO_2_ or N-TiO_2_ were used in the modification, the peak current increased and the GCE/MWCNT/N-TiO_2_ electrode presented the lowest overpotential (~0.05 V for BEZ and ~0.2 V for L-dopa). The results suggest that the nanocomposite with MWCNT and N-TiO_2_ nanoparticles can effectively enhance the conductivity of the working electrode and catalyze the oxidation process of the analytes, which is attributed to reducing the electron transfer resistance, and also enhancing the electrode surface area.

**Figure 5:**
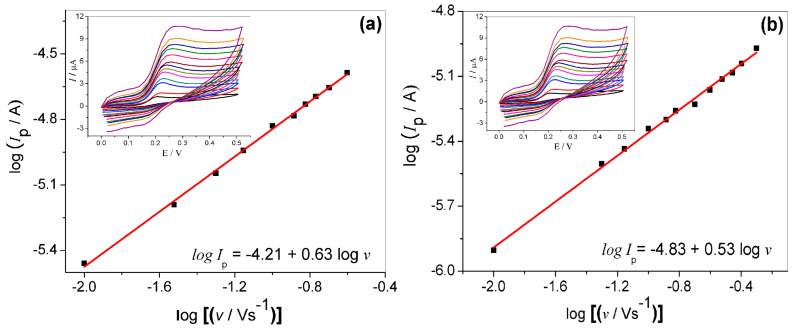
Log (I_p_) vs. log (*v*) plots for 0.5 μmol L^−1^ BEZ (**a**) and 0.1 μmol L^−1^ L-dopa (**b**) obtained from CV experiment (inset) at different scan rates (10–500 mV s^−1^) in 0.1 mol L^−1^ phosphate buffer solution (pH 7.0).

The electrochemical behavior of GCE/MWCNT/N-TiO_2_ was studied at different scan rates (from 10 to 500 mV s^−1^) in the presence of BEZ and L-dopa, as depicted in the inset of Figure 5. The influence of scan rate in the peak current intensity provides an indication of which process is involved in the oxidation of the drugs, diffusion, or charge transfer process. It is noted that the overpotential is shifted positively, which is characteristic of an irreversible process. In accordance to the literature [34], in the electrochemical process whose reaction is controlled by adsorption, the slope of log I_p_ vs. log ν is near 1.0, while in the process controlled by diffusion, the slope is about 0.5. Thus, as noted in Figure 5a,b, the electrode exhibits the linear behavior for both analytes, which presents the slope of 0.63 and 0.53 for BEZ e L-dopa, respectively. For both, in accordance with the results, we can infer that the oxidation mechanism for both BEZ and L-dopa follows a diffusion-controlled process of species from the solution to the electrode surface.

### 2.4. Simultaneous Determination of BEZ and L-Dopa at GC/MWCNT/N-TiO_2_ Electrode

**Figure 6:**
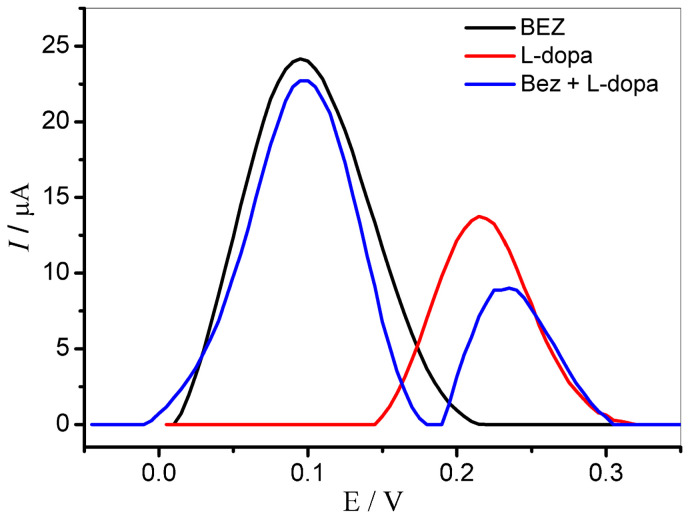
Square wave voltammograms obtained for BEZ (0.2 μmol L^−1^), L-dopa (0.2 μmol L^−1^), and the mixture of both (BEZ + L-dopa 0.2 μmol L^−1^) using a GCE/MWCNT/N-TiO_2_. Supporting electrolyte: 0.1 mol L^−1^ phosphate buffer (pH 7.0). Parameters: *f* = 30 Hz, *a* = 40 mV, and Δ*Es* = 2 ms.

To appraise the possibility of simultaneous determination of BEZ and L-dopa, square wave voltammetry (SWV) technique was used. Figure 6 shows the SW voltammograms obtained for BEZ (0.2 μmol L^−1^), L-dopa (0.2 μmol L^−1^), and the mixture of both analytes.

This study demonstrates that the GCE/MWCNT/N-TiO_2_ is efficient to provide a satisfactory separation for oxidation peaks of BEZ and L-dopa, with a potential difference of 140 mV, clearly allowing the simultaneous determination of the compounds.

**Figure 7:**
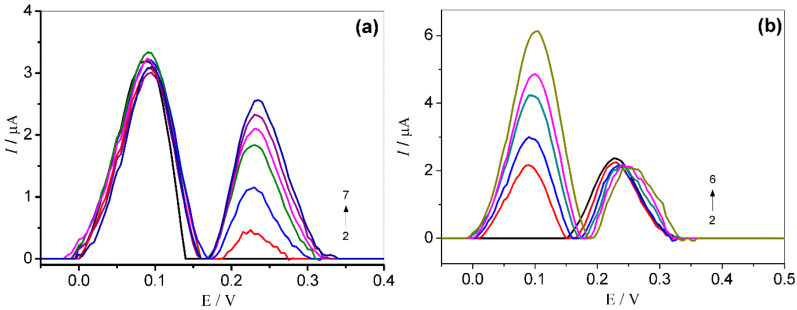
Square wave voltammograms obtained for: (**a**) L-dopa (2–7) 2.0 × 10^−6^–3.0 × 10^−5^ mol L^−1^ and BEZ (1–7) 5.0 × 10^−6^ mol L^−1^ (fixed); (**b**) BEZ (2–6) 2.0 × 10^–6^–8.0 × 10^–6^ mol L^−1^ and L-dopa (1–6) 4.0 × 10^–5^ mol L^−1^ (fixed), using GCE/MWCNT/N-TiO_2_. Supporting electrolyte: 0.1 mol L^−1^ phosphate buffer (pH 7.0). Parameters of SWV: *f* = 30 Hz, *a* = 40 mV e Δ*Es* = 2 ms.

The separate determination of L-dopa in the concentration range 2.0 × 10^−6^–3.0 × 10^−5^ mol L^−1^ was accomplished in solutions containing BEZ at the fixed concentration of 5.0 × 10^−6^ mol L^−1^ (see Figure 7a). As can be seen, the peak oxidation current for L-dopa regularly increases as its concentration is increased at a fixed concentration of BEZ. Similarly, as shown in Figure 7b, the peak oxidation current for BEZ increases regularly as its concentration is increased at a fixed concentration of L-dopa 4.0 × 10^–5^ mol L^−1^.

The results obtained confirmed that the oxidation of both analytes is independent, and simultaneous determination is possible without interference, even at high concentrations.

**Figure 8:**
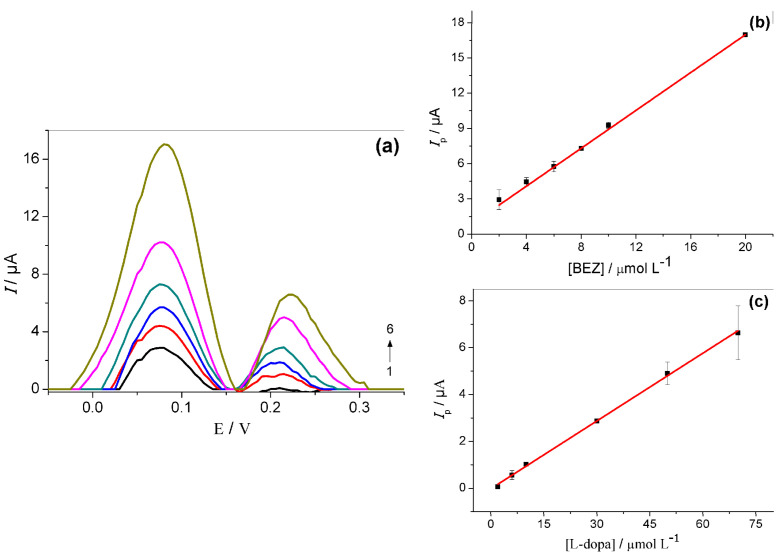
(**a**) SW voltammograms obtained for BEZ (1–6: 2.0; 4.0; 6.0; 8.0; 10.0 e 20.0 μmol L^–1^) and L-dopa (1–6: 2.0; 6.0; 10.0; 30.0; 50.0 e 70.0 μmol L^–1^), using the GCE/MWCNT/N-TiO_2_. Analytical curves obtained for BEZ (**b**) and L-dopa (**c**). Electrolyte: 0.1 mol L^−1^ phosphate buffer (pH = 7.0). Parameters of SWV: *f* = 30 Hz, *a* = 40 mV e Δ*Es* = 2 ms.

After this previous study, both analytes were determined by simultaneously changing their concentrations. SWV voltammograms were carried out from successive and simultaneous addition at concentrations of L-dopa (2.0–70 µmol L^−1^) and BEZ (2.0–20 µmol L^−1^) in 0.1 mol L^−1^ of phosphate buffer, as shown in Figure 8a. The peak current obtained for BEZ and L-dopa has a linear dependence on the respective concentration. The corresponding analytical curves are displayed in Figure 8b,c.

The detection of limit (LOD), which was estimated by the concentration whose associated amperometric response was equal to three times the average voltammetric response for the blank solution (*n* = 10) [35] and other analytical parameters, are shown in Table 1.

Repeatability essays, intra-day and inter-day, were also carried out to ensure the precision of the proposed method. The intra-day repeatability was estimated by ten successive measurements (*n* = 10), while de inter-day essay was estimated by measurements on five successive days (*n* = 5) using the same modified electrode. The corresponding relative standard deviation (RSD) for each analyte is depicted in Table 1. It is worth noting that the values found confirm that the present method has a satisfactory precision for the quantification of BEZ and L-dopa throughout a wide concentration range. The obtained detection limits for BEZ and L-dopa were better than most of the other works in the literature in the case of simultaneous L-dopa and BEZ determination (see Table 2).

As aforementioned, the main advantage of the modified electrode used in the present work is the presence of TiO_2_ nanoparticles doped with N associated with the MWCNT. The heterojunction of these structures can enhance the conductivity of the composite and provide a high sensitivity.

The selectivity of the proposed method was evaluated by the addition of possible interferents (lactose, starch, povidone) to a standard solution containing BEZ and L-dopa at the concentration ratios (standard solution: interferent) 10:1, 1:1, and 1:10 and the obtained current signals were compared with those obtained with the standard solution. The analysis of the obtained responses allowed concluding that these compounds do not significantly interfere with the here-proposed methods using the GCE/MWCNT/N-TiO_2_.

### 2.5. Simultaneous Determination of BEZ and L-Dopa in Pharmaceutical Samples

Finally, to validate the proposed method, BEZ and L-dopa were determined in two different commercial pharmaceutical samples by the standard addition method. Table 3 presents the BEZ and L-dopa concentrations determined simultaneously in the analyzed samples employing the proposed SWV method and an HPLC method [9]. By analyzing the results obtained, one can conclude that the values obtained by the proposed method agree quite well with those obtained by the comparative HPLC method. Applying the paired t-test to the results obtained by both methods, the resulting t values (0.128 for BHA and 0.232 for BHT) are smaller than the critical one (2.31, α = 0.05), indicating that there is no difference between the obtained results, at a confidence level of 95%.

Thus, we can infer that the proposed method using GCE/MWCNT/N-TiO_2_ electrodes is efficient in the simultaneous determination of BEZ and L-dopa.

## 3. Materials and Methods

### 3.1. Apparatus

Cyclic and square wave voltammetry (CV and SWV) experiments were performed using a PalmSens 2 (PalmSens) potentiostat/galvanostat controlled with the PSTrace 5.8. The SW voltammograms were baseline-corrected by the moving average method (peak width: 0.003). The electrochemical impedance spectroscopy (EIS) studies were carried out using an Autolab PGSTAT 30 potentiostat/galvanostat with FRA module controlled by Nova 1.8 software. A one-compartment three-electrode glass cell system was used for the electrochemical measurements, with a GCE modified or not with MWCNT/N-TiO_2_ (GCE/MWCNT/N-TiO_2_) as a working electrode, Pt wire as an auxiliary electrode, and an Ag/AgCl (3.0 mol L^−1^ KCl) as reference electrode, hereinafter, all potentials are referred to this reference electrode. All pH measurements were made on a Mettler Toledo digital pH meter fitted with a glass electrode as working and an Ag/AgCl reference electrode, which was previously standardized with buffer solutions of known pH. A DL-180 ultrasonic apparatus was used for sonicating the suspension of the composites.

The BEZ and L-dopa determinations by HPLC were carried out using an LC-10 AT Shimadzu system, with an ultraviolet–vis detector (SPD-M10-AVP) set at 180 and 210 nm. A Shim-Pack CLC-ODS (6.0 × 250 mm, 5 µm) chromatographic column was used. The procedure was developed by Wollmer and Klein [9] using a gradient elution with a mobile phase consisting of solvent A: 30 mmol L^−1^ phosphate buffer pH 2.50, and the pH of the solvent was adjusted with orthophosphoric acid. Solvent B: A mixture of water that, prior to mixing, had been adjusted to pH 2.50 with orthophosphoric acid and acetonitrile (50:50, *v*/*v*). The flow rate was 1.5 mL min^−1^, and the injection volume was 30 µL.

### 3.2. Reagents, Supporting Electrolytes, and Standards

All reagents were of analytical grade. L-dopa (99%) and BEZ (98%) were purchased from Sigma (St. Louis, MI, USA). Aqueous phosphate Buffer solution, 0.10 mol L^−1^ pH 7 (which was prepared using adequate amounts of NaH_2_PO_4_ and Na_2_HPO_4_), was used as a supporting electrolyte. The 0.10 mol L^−1^ L-dopa or BEZ stock solution was prepared in the supporting electrolyte, from which appropriate aliquots were diluted. All solutions were prepared using ultra-purified water (resistivity > 18 MΩ cm) supplied by a Milli-Q system (Millipore ^®^ São Paulo, Brazil).

### 3.3. Synthesis of N-Doped TiO_2_

The synthesis of N-doped TiO_2_ nanoparticles was realized by the supercritical antisolvent precipitation (SAS) method, according to the modified procedure described by Silva et al. [37]. In summary, two solutions (125 mL each) were injected into an expansion chamber with a continuous flow of 5 mL min^−1^. The first solution is composed of 0.5 mol L^−1^ of TIP in isopropanol, and the second solution composed of water/ethanol (22% *w*/*w*) and ammonium hydroxide (5.4 mol L^−1^). CO_2_ was pumped perpendicularly to the chamber at the continuous flow of 40 g min^−1^. After, the chamber was kept under pressure of 150 bar at 40 °C during the injection of both solutions. Thereafter, doped TiO_2_ nanoparticles were dried still in the precipitation chamber at 40 °C under a constant flow of 40 g·min^−1^ of CO_2_ for 240 min. Then, the final product was collected and annealed at 450 °C for 2 h.

### 3.4. MWCNTs Treatment and Preparation of GCE/MWCNT/N-Doped TiO_2_

Previously, MWCNTs were functionalized by acid treatment according to what was described in the literature. The modified electrodes were prepared by a simple casting method. Prior to the surface coating, the GC electrode was polished on a polishing cloth with alumina powder. Then the electrode was cleaned by ultrasonication in ethanol and deionized water, respectively. To obtain the modified electrode, 1.0 mg of MWCNT and 1.0 mg of N-doped TiO_2_ and/or TiO_2_ were dispersed in chitosan solution (1% *m*/*v*) prepared in acetic acid solution (1% *v*/*v*). The dispersion resultant was placed in an ultrasonic bath for 1 h. Thus, 10 µL of the dispersion were dropped on GCE surface. The modified electrodes were labeled as GCE/MWCNT, CGE/MWCNT/TiO_2_, GCE/N-TiO_2_, and GCE/MWCNT/N-TiO_2_.

### 3.5. Preparation of Pharmaceutical Formulations

The proposed method was carried out for the simultaneous determination of L-dopa and BEZ in pharmaceutical formulations. Two commercial samples of pharmaceutical formulations (200 mg of L-dopa and 50 mg of BEZ) were purchased in a local market. Ten tablets of each analyzed pharmaceutical formulation were accurately weighed and finely powdered in a mortar, transferred into a calibrated flask, and completed to the volume with phosphate buffer pH 7 to prepare a solution a stock solution. After that, an appropriate aliquot was diluted with the same supporting electrolyte. The pharmaceutical formulation samples were analyzed using the standard addition method, and the L-dopa and BEZ contents found were compared with the label values.

### 3.6. Morphology Characterization and Measurement Procedures

The morphology of MWCNT/N-TiO_2_ nanocomposite was examined using a transmission electron microscope (TEM)—Jeol model JEM 2100 operating at an accelerating voltage of 120 kV. All electrochemical measurements were carried out using a 10 mL electrochemical cell at room temperature (25 ± 1 °C). The deaeration of the supporting electrolyte was not necessary since no interferences from O_2_ were detected under the studied experimental conditions.

The EIS experiments were performed at the formal potential of the [Fe(CN)_6_]^4−/3−^ redox couple, from 10 mHz to 100 kHz (10 points per decade) and with a 10 mV (r.m.s.) ac perturbation, for a 1.0 mmol L^−1^ K_4_Fe(CN)_6_ in 0.5 mol L^−1^ KCl solution.

CV and SWV were employed to investigate the electrochemical behavior and the simultaneous quantification of L-dopa and BEZ. The instrumental parameters for SWV were optimized, and the respective analytical curve was obtained by adding small volumes of concentrated standard solutions of the L-dopa and BEZ to the supporting electrolyte solution. The limit of detection (LOD) values were calculated as three times the standard deviation for 10 measurements of the blank solution (*s*) divided by the slope of the respective analytical curve (b) (LD = 3*s*/b) [35]. The repeatability of the electroanalytical methods was checked with intra-day (*n* = 10) and inter-day (*n* = 5) determinations for two different concentrations of L-dopa and BEZ, for which the respective relative standard deviations (RSD) were calculated.

The selectivity of the proposed methods was evaluated by the addition of possible interferents present in pharmaceutical formulations and urine samples (uric acid, urea, lactose, starch, povidone) to a standard solution containing L-dopa and BEZ, in the concentration ratios (standard solution to interferent) of 10, 1, and 0.1.

## 4. Conclusions

In this work, we successfully developed a novel electrochemical method based on the glassy carbon electrode modified with MWCNTs and N-doped TiO_2_ nanoparticles. We explored the high electroactive surface area of MWCNTs and the high conductivity of nitrogen doping TiO_2_ nanoparticles. Hence, the synergic effect between the materials led to an excellent separation of oxidation peak potential (140 mV), allowing the simultaneous determination of BEZ and L-dopa. Moreover, good sensitivity and low LOD (1.6 μmol L^−1^ for BEZ and 2.0 μmol L^−1^ for L-dopa) were achieved. Finally, the application of the developed method is very suitable for accurate determinations of BEZ and L-dopa in the pharmaceutical samples with a relative error lower than 3%. Furthermore, the proposed methods are simple, quick, and can be carried out with good precision and accuracy. It can be a less expensive alternative for application in routine determinations of these drugs.

## Figures and Tables

**Table 1:** Analytical parameters obtained for BEZ and L-dopa by the proposed electroanalytical method.

Parameters	BEZ	L-Dopa
Oxidation Potential (V)	0.08	0.21
Linear concentration range (μmol L^−1^)	2.0–20	2.0–70
Correlation coefficient (r)	0.999	0.998
Slope (μA·μmol L^−1^)	0.792	0.0958
Intercept (μA)	1.18	−0.0106
LOD (μmol L^−1^)	1.6	2.0
Repeatability of peak current—Intra-day (RSD % ^1^)	1.1	5.8
Repeatability of peak current—Inter-day (RSD % ^1^)	1.9	7.9

^1^ Relative Standard Deviation.

**Table 2:** Comparison of results obtained for the simultaneous determination of BEZ and L-dopa by the here-proposed method and by other electrochemical methods reported in the literature.

Electrode	Techniques	Supporting Electrolyte	Linear Concentration Range (μmol L^−1^)		LOD (μmol L^−1^)		Reference
			BEZ	L-Dopa	BEZ	L-Dopa	
GCE ^1^	DPV	HClO_4_			2.77	5.12	[10]
PAR-GCE ^2^	DPV	PBS (pH 5.2)	10–200	25–1000	2.00	6.00	[11]
Poly(3-MT)-MWCNT-COOH/GCE ^3^	DPV	PBS (pH 7.0)	400–1000	50–95	32.5	32.3	[12]
SPCE electro-chemically pretreated ^4^	DPV	BRB (pH 2.21)	6–100	1–100	2.6	0.47	[36]
GCE/MWCNT/N-TiO_2_	SWV	PBS (pH 7.0)	2–20	2–70	1.6	2.0	This Work

^1^ GCE: Glassy Carbon Electrode; ^2^ PAR-GCE: Poly(4-(2-pyridylazo)-resorcinol) (PAR) modified Glassy Carbon Electrode; ^3^ Poly(3-MT)MWCNT/GCE: Electropolymerized Poly(3-methylthiophene (MT)) and Functionalized Carboxylated Multi-Walled Carbon Nanotube Modified Glassy Carbon Electrode; ^4^ SPCE electro-chemically pretreated: Electro-chemically Pretreated Screen-Printed Carbon Electrode.

**Table 3:** Results obtained for Benserazide (BEZ) and Levodopa (L-dopa) determination in commercial pharmaceutical samples by the SW voltammetric proposed method and high-performance liquid chromatography (HPLC) comparative method.

	Label		BEZ and L-Dopa Amount (mg/tablet) ^a^				Relative Error ^b^ (%)	
			SWV Proposed Method		HPLC Proposed Method			
Sample	BEZ	L-Dopa	BEZ	L-Dopa	BEZ	L-Dopa	BEZ	L-Dopa
A	50	200	52 ± 4	198 ± 5	51 ± 1	202 ± 3	1.9	−2.0
B	50	200	51 ± 2	203 ± 6	53 ± 2	199 ± 2	−3.8	2.0

^a^ average of 3 measured concentrations; ^b^ 100 × ([SWV method − HPLC method]/HPLC method).

## Data Availability

Not applicable.
